# Obesity and insulin resistance are associated with reduced activity in core memory regions of the brain

**DOI:** 10.1016/j.neuropsychologia.2017.01.013

**Published:** 2017-02

**Authors:** Lucy G. Cheke, Heidi M. Bonnici, Nicola S. Clayton, Jon S. Simons

**Affiliations:** Department of Psychology, University of Cambrigde, UK

**Keywords:** Obesity, Episodic Memory, Insulin resistance, What-where-when, FMRI

## Abstract

Increasing research in animals and humans suggests that obesity may be associated with learning and memory deficits, and in particular with reductions in episodic memory. Rodent models have implicated the hippocampus in obesity-related memory impairments, but the neural mechanisms underlying episodic memory deficits in obese humans remain undetermined. In the present study, lean and obese human participants were scanned using fMRI while completing a What-Where-When episodic memory test (the “Treasure-Hunt Task”) that assessed the ability to remember integrated item, spatial, and temporal details of previously encoded complex events. In lean participants, the Treasure-Hunt task elicited significant activity in regions of the brain known to be important for recollecting episodic memories, such as the hippocampus, angular gyrus, and dorsolateral prefrontal cortex. Both obesity and insulin resistance were associated with significantly reduced functional activity throughout the core recollection network. These findings indicate that obesity is associated with reduced functional activity in core brain areas supporting episodic memory and that insulin resistance may be a key player in this association.

## Highlights

• Obesity associated with reduced activity in core recollection network during episodic memory.
• Insulin resistance associated with reduced activity in core recollection network during episodic memory.
• Insulin resistance, but not obesity, associated with poorer memory performance.

## 1 Introduction

Obesity is a major risk factor for premature mortality (Kopelman, 2000) and carries an enormous financial burden for health care providers worldwide (Gorsky et al., 1996). With nearly half of the US population currently overweight or obese (World Health Organization, 2010), and prevalence rising, understanding the neurobiological correlates of this condition is becoming increasingly important.

There is a growing literature exploring the association between obesity and cognitive health (Prickett et al., 2015). A number of studies have demonstrated a negative association between anthropometric measures of obesity, such as body weight, body mass index (BMI), or waist circumference (WC), and cognitive performance (Elias et al., 2012), in particular worse executive function (Barkin, 2013, Gunstad et al., 2007). However, other studies have found no such association (van Boxtel et al., 2007) or even small positive associations with cognition (Kuo et al., 2006). One particular cognitive domain which has been suggested as displaying an impairment in obesity is episodic memory. There is considerable evidence for reduced memory performance in rodent models of obesity (Jurdak et al., 2008, Popovic et al., 2001). Obesity in humans has been associated with poor performance on measures of verbal learning such as delayed recall and recognition (Cournot et al., 2006, Gunstad et al., 2006) as well as visual what-where-when episodic memory tasks (Cheke et al., 2016). However, again this association with memory is not seen in all studies (Conforto and Gershman, 1985, Nilsson and Nilsson, 2009).

The mixed results in the behavioural literature are reflected in imaging investigations. Willette and Kapogiannis (2015) reviewed articles that directly or indirectly addressed the association between adiposity (most commonly defined by BMI) and brain volume. A general trend towards lower global gray-matter volume was found in individuals of all ages. In particular, a negative association between BMI and volume in the prefrontal cortex was found in 17/23 studies assessing that area (Maayan et al., 2011, Pannacciulli et al., 2006, Smucny et al., 2012, Ursache et al., 2012, Weise et al., 2013) although 1 study showed an association in the other direction (Taki et al., 2008). Results from the temporal lobe were more mixed; 14/22 studies to investigate temporal lobe volume found increased atrophy with adiposity (Brain Development Cooperative Group, 2012; Pannacciulli et al., 2006; Weise et al., 2013) while others did not, and only 11/28 of the studies specifically investigating hippocampal volume found a negative association with obesity (Anan et al., 2010, Bruehl et al., 2011, Debette et al., 2010, Ho et al., 2011, Ho et al., 2010a, Ho et al., 2010b, Jagust et al., 2005, Kurth et al., 2013, Taki et al., 2008) with two studies reporting associations in the other direction (Kurth et al., 2013, Widya et al., 2011).

There has, to date, been only one functional imaging investigation of episodic memory in obesity. Boraxbekk et al. (2015) assigned 20 overweight middle-aged women to either a modified Palaeolithic diet or a standard healthy diet for 6 months. The authors assessed episodic memory with a face-name recognition paradigm while using functional magnetic resonance imaging to examine brain activity before and after the dietary intervention. Memory performance improved significantly after the dietary interventions (with no difference between the two groups), and decreases in waist circumferences correlated significantly with increased brain activity in the superior temporal gyrus and insula. However, the results of this study are difficult to interpret for a number of reasons. First, a recognition face-name paradigm may rely on a number of mnemonic processes and cannot be said to be specifically assessing episodic memory. Second, due to the lack of a control group, and because the same memory stimuli were used at the beginning and end of the intervention, it is problematic to determine what degree of change in performance and brain activity one might expect without dietary intervention during this time period.

A major issue in the investigating the association between obesity and cognition is the heterogeneity of the obese population. While “obesity” is defined in terms of excess adiposity, individuals matched for body mass index may vary considerably in comorbid conditions such as hypertension and insulin resistance, and it may be these factors that are mediating the association between obesity and cognition. For example, Gonzales et al. (2010) assessed the impact on obesity on functional activity during a 2-back working memory task in 32 cognitively normal middle aged adults with BMI's ranging from the healthy range to obese. In addition, the authors examined insulin sensitivity as a potential factor mediating the association between BMI and brain activity. It was found that the obese group displayed significantly lower task-related activity in the right parietal cortex (BA 40/7) than either the normal or overweight group; an effect that was found to be fully mediated by insulin sensitivity.

It has been well established that obesity increases the risk of insulin resistance and type 2 diabetes mellitus (Bonadonna et al., 1990, Matsuzawa et al., 2011). Diabetes currently affects around 250 million people worldwide (Cole et al., 2007) and is particularly prevalent in older adults (Wild et al., 2004). While the systemic damage caused by diabetes is well described (Zhao et al., 2010), only recently have researchers began to recognise the significance of insulin and insulin resistance for brain and cognitive health.

Insulin is a peptide released by pancreatic cells, which has multiple functions both in the periphery and in the central nervous system. Whether insulin is synthesized in the adult brain is a topic of controversy, however it is known to readily cross the blood brain barrier and perform many important functions within the brain (Abbott et al., 1999, Banks et al., 1997, Baskin et al., 1987, Baura et al., 1993, Chiu et al., 2008; Zhao and Townsend, 2009). Insulin receptors appear in high concentrations in the cerebral cortex and hippocampus (Baskin et al., 1987, Havrankova et al., 1978a, Havrankova et al., 1978b, Lathe, 2001, Unger et al., 1991), and there is substantial co-localisation for insulin-containing neurons, insulin receptors and glucose transporter isoforms in the hippocampus and medial temporal lobe (Grillo et al., 2009). There are many proposed mechanisms by which insulin may modulate learning and memory: Examples include stimulating glucose uptake in key regions (Grillo et al., 2009), modulating expression of NMDA in the cell membrane, affecting the induction of long-term potentiation (LTP; Skeberdis, Lan, Zheng, Zukin, and Bennett, 2001) and modulating CNS levels of acetylcholine and norepinephrine (Figlewicz et al., 1993, Kopf and Baratti, 1996).

While the exact mechanisms by which insulin influences learning and memory remain to be fully elucidated, a number of lines of evidence suggest that changes in insulin levels and/or regulation can have significant consequences for cognition. The impact of insulin administration on declarative memory has been investigated in human and animal models and reliably shows a beneficial effect. In rats, intracerebroventricular administration of insulin improves performance on passive avoidance tasks (Park et al., 2000), while intranasal insulin administration has resulted in improved performance on water maze and radial arm tasks (Francis et al., 2008). In humans, intravenous infusion of insulin (while keeping glucose levels stable) has been found to improve hippocampal-dependant (word-list) memory (Kern et al., 1999), and such findings have been reflected in trials using intranasal infusions of insulin (Benedict et al., 2004, Benedict et al., 2008, Hallschmid et al., 2008, Stockhorst et al., 2004). For example, Benedict and colleagues demonstrated that declarative memory (as measured by word list paradigms) could be improved in young healthy adult subjects by means of an 8-week course of intranasal insulin administration.

Insulin resistance (IR) can be broadly defined as a reduced cellular responsiveness to insulin (Goldstein, 2002), characterized by higher insulin levels needed to maintain glucose levels in the periphery and brain. Growing evidence has linked insulin resistance to cognitive decline and neurodegeneration (e.g. Craft et al., 2013). Higher IR in middle aged adults is a mediator for worse memory performance and greater reduction in GM volume over a 4-year period (Willette et al., 2013) supporting a suggested 7–13% increase in dementia in the presence of type 2 diabetes (Biessels and Kappelle, 2005, Craft et al., 2013, Craft and Watson, 2004, Roriz-Filho et al., 2009, Schrijvers et al., 2010). Indeed, higher levels of insulin resistance markers in MCI and AD patients are associated with worse performance on tests of working and episodic memory independent of plaque and tangle load. These findings suggest that disturbances in insulin signalling has a direct association with cognitive status in older adults (Talbot et al., 2012) rather than acting via increases in beta- amyloid and tangles, although there is also evidence for this route (Cole and Frautschy, 2007). Furthermore, pilot data suggests that intranasal infusions of insulin can be used to improve verbal memory both acutely and chronically in these patients without affecting insulin or glucose in the periphery (Reger and Craft, 2006, Reger et al., 2008a, Reger et al., 2008b).

Long term insulin resistance is a key diagnostic criterion for diabetes mellitus. Both Type 1 and Type 2 diabetes are associated with reduced memory and executive functions (Awad et al., 2004, Grodstein et al., 2001, Kodl and Seaquist, 2008, Messier, 2005, Munshi et al., 2006, Perlmuter et al., 1984, Weinger et al., 2008). Children with type 1 diabetes demonstrate worse school performance and IQ scores than their nondiabetic peers (Dahlquist et al., 2007, Fox et al., 2003, Northam et al., 2001, Schoenle et al., 2002) and these impairments appear to persist into adulthood (Ryan, 2006). Structural neuroimaging studies suggest that individuals with type 1 and type 2 diabetes demonstrate cortical and subcortical atrophy, including the hippocampus and amygdala, related with impaired cognitive performance (Akisaki et al., 2006, Dejgaard et al., 1991, den Heijer et al., 2003, Longstreth et al., 1998, Perantie et al., 2007). These findings were significant even after controlling for cardiovascular health. Indeed, there was a strong interaction between diabetes and hypertension such that individuals exhibiting both conditions were at several times greater risk than those with either condition alone (Lobnig et al., 2006). Finally, cognitive decline and neurodegeneration has been shown to be increased not only in diabetes itself, but in pre-diabetes (Luchsinger et al., 2004), in which there is insulin resistance but enough insulin is still produced to prevent overt diabetes (Cole et al., 2007; Luchsinger et al., 2004; Yaffe et al., 2004).

To date, the vast majority of studies in this area have been conducted on older or middle aged adults and as such the association between obesity, insulin resistance and cognitive and neural function in otherwise healthy young adults remains unclear. Furthermore, while a number of studies have examined the impact of obesity and insulin levels on brain areas that previous research indicates to be involved in memory, or a change in brain activity after dietary intervention, none have compared functional activity in these areas during episodic memory task performance in lean and obese individuals. As such, it is currently difficult to assess the relevance of previous neuroscientific findings for understanding memory function in obesity. A direct investigation is therefore needed to understand the neural mechanisms underlying episodic memory deficits in obese individuals. In particular, investigation of group activity differences in core memory areas during both encoding and retrieval of complex episodes can inform as to whether potential impairments may originate during the formation or retrieval of memories, and where in the brain these impairments may be localized. Further, it is important to investigate potential metabolic processes by which such neurocognitive impairments may arise, such as via elevated circulating insulin levels and concomitance insulin resistance, to inform methods by which such deficits can be addressed through intervention or prevention.

Episodic memory involves the encoding, retention and recall of personally experiences episodes, and is often defined in terms of both content, which is temporo-spatial in nature (Tulving, 2002), and the phenomenological experience of vividly “re-living” a particular event. The complex nature of episodic memory means that it is a highly neurally distributed cognitive function, which involves inputs from, and interactions between, areas within the prefrontal, parietal and temporal cortices (e.g. Rugg and Vilberg, 2013; Simons and Spiers, 2003), as well as key subcortical areas such as the hippocampus. While the precise neural underpinnings of episodic memory are yet to be fully defined, different areas within this core recollection network are thought to be particularly important for different elements within memory. For example, the hippocampus is highly involved in both encoding and retrieval of episodic memory (Scoville and Milner, 1957), and is particularly involved in memories that involve a spatial component (e.g. Burgess et al., 2002). Indeed, many have argued that the defining phenomenology of episodic memory relies on the ability of the hippocampus to construct a “scene” in which memories events are played out (e.g. Hassabis and Maguire, 2007). The nature of this “scene” is increasingly thought to rely on areas within the parietal lobe such as the precuneus (Frings et al., 2006) and angular gyrus (Vilberg and Rugg, 2008) which, among other functions, appear to be important for visuospatial imagery (Fletcher et al., 1995) and vividness (Yazar et al., 2014) respectively. The ability to effectively guide encoding and retrieval of appropriate information is largely underpinned by the prefrontal cortex (PFC). For example, areas within the lateral PFC are thought to be important for goal-directed encoding and retrieval specificity (Fletcher and Henson, 2001), while the anterior PFC is thought to be involved in monitoring and evaluation of memory characteristics, particularly object-location associations (Burgess et al., 2001; Ranganath and Paller, 2000).

Cheke and colleagues (2016) used a novel “What-Where-When” (WWW) style episodic memory task (the “Treasure-Hunt task”) in young adults to demonstrate a negative association between bodyweight and memory performance such that higher BMI was associated with poorer performance across item, spatial, temporal, and integrated WWW memory tasks. This form of memory test may be more representative of everyday episodic memory than recognition or word-list tasks as it assesses recollection for multiple aspects of complex episodes beyond item memory alone, as well as the ability to integrate these features into a single temporo-spatial event recollection (Tulving, 2002). In the present study, lean and obese participants matched for age, sex, and IQ completed the Treasure-Hunt task while being scanned using functional magnetic resonance imaging (fMRI). Following Cheke and colleagues’ findings, it was anticipated that the obese group would be significantly impaired at memory for item, spatial, temporal, and integrated WWW features. We hypothesized that these impairments would be associated with reduced functional activity in the hippocampus, prefrontal, and parietal cortices (Raji et al., 2010, Ursache et al., 2012). To investigate the potential mechanisms by which these deficits may arise, blood samples were assessed for circulating levels of insulin, leptin, glucose, and triglycerides. We hypothesized that insulin levels, and particularly a measure of insulin resistance calculated from circulating insulin and glucose, would also have a significant impact on memory and memory-related brain activity.

In summary, this investigation has four central predictions: First, that the Treasure-Hunt task will be associated with increased activity relative to a control task in regions of interest traditionally associated with episodic memory. Second, that obesity will be associated with impaired performance on all aspects of the Treasure-Hunt task. Third, that this impairment will be accompanied by reduced activity in our episodic memory regions of interest, and finally, that insulin resistance will have as large, or larger, impact on memory and memory-related brain activity than BMI.

## 2 Materials and methods

### 2.1 Participants

Thirty-four right-handed participants aged 18–36 were recruited, of which 17 were obese (defined as body mass index [BMI]>30), and 17 lean (BMI 18–25), both groups were 58% female. The data for two participants (1 obese and 1 lean, both female) were excluded due to technical problems (N=1) and structural abnormalities that became evident on the participant's MRI scan (N=1). To investigate behavioural and neural differences associated with the highly related variables of BMI and insulin resistance (IR), it was necessary for the sample to be grouped and analyzed twice, once by BMI and once by insulin resistance. Details of these groupings are shown in Table 1. This regrouping involved 8 individuals (25%) ‘moving’ groups: with the high IR group containing 12 obese and 4 lean, and the low IR group containing 12 lean and 4 obese.

Participants were recruited from the general population through posters and internet advertisement. Potential participants were screened for previous mental health issues, diabetes, use of recreational drugs and MR contra indicators (such as metal in the body or claustrophobia). Basic demographic information was obtained and participants completed the Shipley Institute of Living Scale (SILS) vocabulary test. Using these data, potential recruits to the lean and obese groups were matched for sex, age, years of education and IQ (SILS) before being invited for scanning. The study was approved by Cambridge University Human Biology research ethics committee. Written informed consent was obtained from each participant.

### 2.2 Task

**Fig. 1:**
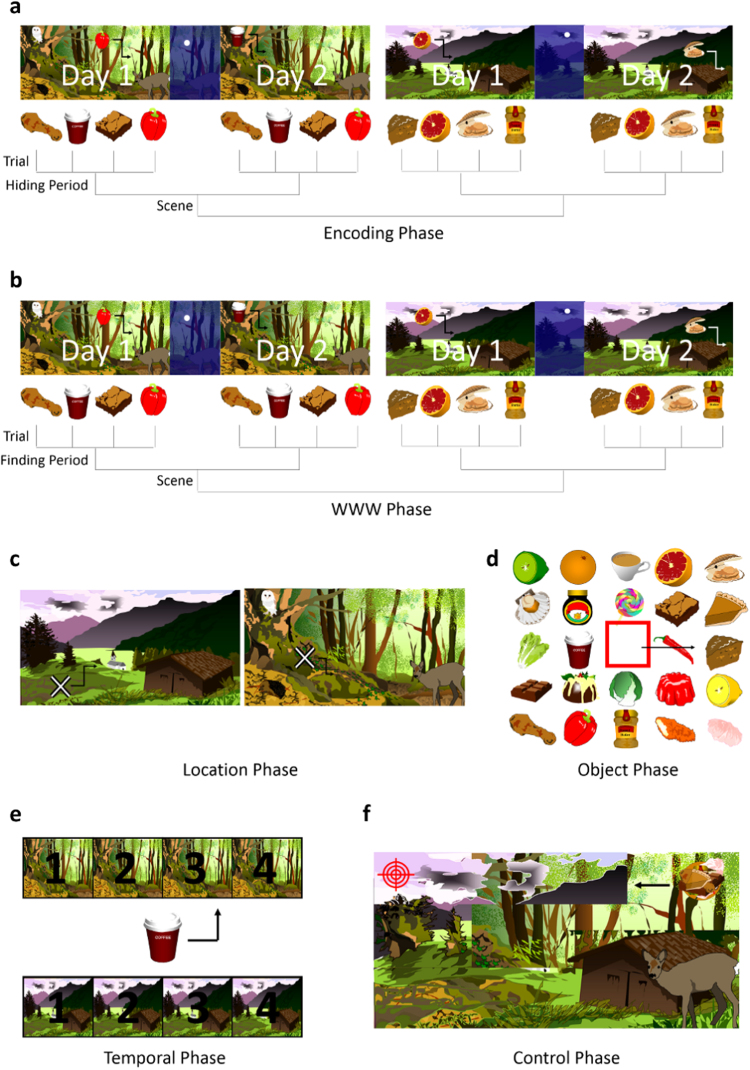
Schematic of Treasure-Hunt Task. Items are moved around in a complex scene or response menu. a) Encoding b) What-Where-When retrieval c) Location retrieval d) Object retrieval e) Temporal retrieval f) Control task.

The Treasure-Hunt task was programmed using Psychopy (Peirce, 2008). Each session of the task contained 6 phases, beginning with a single encoding phase, followed by four retrieval phases and a control phase administered in a random order (see Fig. 1). Each trial of each task lasted 7.5 s. If participants did not finish a trial within 7.5 s a time-out symbol (stop sign) would appear briefly before the commencement of the next trial. If participants completed a trial in less than 7.5 s a signal would appear (a cross for the encoding, WWW and temporal tasks, the cursor changing color in the object and location tasks) for the remaining time until the commencement of the next trial.

The encoding phase consisted of four “hiding periods” in each of which participants were instructed to move 4 food items (e.g., an apple, a donut, an aubergine) around in a complex scene (e.g., a forest, a beach, a desert) and “hide” them in locations of their choice. The encoding phase contained two scenes and each item was hidden twice within a given scene, across two hiding periods, clearly labelled “day 1” and “day 2” which occurred consecutively, separated by a 2 s “night” scene (see Fig. 1). As such participants hid 4 items in scene 1, day 1; then the same 4 items in new locations in scene 1 d 2, then 4 new items in scene 2 d 1 and then the same 4 items in new locations in scene 2 d 2. This resulted in a total of 16 item-location-time combinations which overlapped in particular features but were each a unique combination.

The four retrieval phases consisted of What-Where-When (WWW) memory, location memory, temporal memory, and object memory. The WWW memory phase took the exact same form as the encoding phase, with four studied items appearing at random locations in the complex scene in which they were previously encountered on either “day 1” or “day 2”. Participants were instructed to move each item to the location in the scene in which they had hidden it on that “day”. In the location memory phase, a cross appeared at a random position in the scene. Participants were instructed to move the cross to a location where they “had hidden something”. They were not required to indicate what item they had hidden there or on which “day” they had hidden it. In the object memory phase, participants were presented with an array of 24 items, 8 of which were the items participants had hidden during the encoding phase, whereas 16 items were distractors which were loosely matched for semantic and perceptual similarity (e.g. a cucumber vs. cougette, Oreo vs. chocolate digestive). Distractor items were “pure” distractors, in that they did not appear as target items in any other task or trial, and different distractors were used for each session. For each trial, participants were required to move a red square-shaped cursor over an item which they had previously hidden, and select that item. In the temporal phase, participants were presented with four icons for each scene, numbered 1–4. An item appeared in the centre of the screen and participants were instructed to move the item to the appropriate scene icon to indicate the order in which they had hidden that item. For example, if the cucumber was the 4th item hidden in the desert scene, it should be moved to the desert icon labelled with a “4”. Finally, the control phase was designed to match the other tasks in terms of visual and motor processing, but not involve memory. This task involved a melded image of the two scenes used in that session, and a melded item constructed from all of the items used in that session. The melded item would appear in random locations on the melded scene. At the same time, a target would appear at another random location in the scene. Participants were required to move the melded item to the target. 8 trials of the control task were conducted for each session.

Participants completed four full sessions of the Treasure-Hunt task, each comprising unique scenes and items, therefore completing 64 trials each of encoding, WWW, and location tasks, and 32 trials each of the object, temporal and control tasks. As such, the encoding, WWW, and location tasks lasted twice as long as the object, temporal and control tasks. Across these four sessions, all tasks, including the control, were presented in a different random order, with the exception of the encoding task that always occurred at the beginning of the session.

### 2.3 Coding

Accuracy in the WWW and location tasks was assessed according to the distance between the location in which the correct item from the correct day (WWW) or any item (Location) was hidden during encoding and the location indicated during retrieval. For behavioural analysis, only completely accurate answers were coded as correct, allowing the detection of subtle individual differences in memory accuracy. However, in order to provide sufficient trials for a well powered imaging analysis, a more generous success criterion was adopted for the purposes of event-related fMRI analysis. Here, participants were considered correct if they were fewer than 2 button-presses away from the target. The format of this task also allowed investigation of the types of errors individuals made in the WWW task. Where individuals indicated a completely wrong location, these were labelled “spatial errors”, where the correct location and item was indicated, but for the wrong “day” (e.g. correctly identifying the day-2 position when they were asked to report the day-1 position) these were labelled “session errors”. Finally, where the correct location for the correct day but for the wrong object was indicated, these were labelled “item errors”. Accuracy in the temporal and object tasks was coded according to whether participants indicated the correct object or icon.

### 2.4 Imaging data acquisition

Structural MPRAGE images and functional images were acquired with a 3T TIM TRIO Scanner (Siemens, Germany) (repetition time=2440 ms, echo time=30 ms, 41 interleaved axial slices oriented at 10–20 degrees from the AC-PC transverse plane, 2 mm thickness, 1 mm interslice skip, 192 mm field of view [FOV], 64×64 matrix). In order to allow for T1 equilibration, the first 6 functional volumes from each session were discarded. Data were pre-processed and analyzed using SPM8 (Wellcome Trust Centre for Neuroimaging, UCL, London). To correct for distortion (Hutton et al., 2002), field maps were acquired with a standard magnetic field mapping sequence (TE=5.19 and 7.65 ms, TR=400 ms, matrix size=64×64) using 32 slices covering the whole head (voxel size 3×3x3 mm). All acquired images for each participant were realigned with respect to the first for motion correction and all slices were resampled in time to match the middle slice. Participants’ structural scans were coregistered to their mean functional image, and the coregistered structural scan was segmented to separate out gray matter and generate normalization parameters. Next, these normalization parameters were used to normalize the realigned and slice-timing corrected functional images into 3×3×2 mm voxels in Montreal Neurological Institute (MNI) stereotactic space (Cocosco et al., 1997). The normalized images were then spatially smoothed with an 8 mm full- width at half-maximum isotropic Gaussian kernel.

All analyses used an event-related design. Statistical analysis of random effects was undertaken in 2 stages. In the first stage, event types for each session were modelled by convolving the onset times of trials associated with correct retrieval with a canonical hemodynamic response function. Low frequency noise was removed with the use of a 1/128 Hz high pass filter and an AR(1) model corrected for temporal autocorrelation. A subject- level model was used to estimate the parameters for each regressor, with movement parameters in the 3 directions of motion and 3 degrees of rotation included as vectors of no interest to avoid movement confounds. For the Treasure-Hunt task, 6 separate regressors (duration=0) coded the onsets of: 1) Encoding, 2) WWW retrieval, 3) Location retrieval, 4) Temporal retrieval, 5) Object retrieval, and 6) control trials. For the retrieval tasks, only trials in which the behavioural response was correct were included, this was 13% of WWW and location memory trials, 8% of temporal memory trials and 21% of object memory trials. At the second level, the resulting contrast estimates (Encoding-Control, WWW-Control, Location-Control, Temporal-Control, Object-Control, and Control) were entered into a group level factorial GLM. SPM T-images were estimated for each paired condition comparison, treating subject as a random effect. The contrasts between task and control were taken to a group- level ANOVA. This was run with task-type and either IR-group or BMI-group as factors.

Coordinates of the brain regions of interest (ROIs) were identified from previous literature investigating episodic memory (see Table 2) with particular emphasis on areas sensitive to the encoding and/or retrieval of object-location associations and including a control area (the vertex) for which there is no established role in memory. Where possible, these coordinates were taken from meta-analyses, so as to reduce sensitivity to noise (Poldrack, 2007). These were used to create 10 mm diameter spherical masks for small-volume correction. Activations within these regions were reported as significant if the peak exceeded the small volume corrected family-wise error threshold of P<0.05. Beta values for these ROIs were extracted and correlated with memory performance and levels of circulating hormones.

### 2.5 Procedure

Eligible participants were invited to attend scanning at the Wolfson Brain Imaging Centre at 8 A.M., having fasted overnight. On arrival, they completed a practice version of the Treasure-Hunt task using the 5-button box that they would use in the scanner. They were given explicit instructions not to hide any two items in the same location, and to use the landmarks of the scene, rather than the geometry of the screen when hiding items. Scanning commenced at 8.30 A.M. and lasted for one hour. After scanning, participants provided a blood sample and completed the Ravens progressive matrices task (Advanced, set II). Finally, participants were weighed and had their height measured (to calculate BMI), paid £30 and given a picture of their brain scan for participating.

### 2.6 Blood samples and hormone analysis

Participants were instructed to fast overnight before attending the study. After scanning, participants provided a 2 ml blood sample. These were immediately centrifuged and the plasma frozen at −80°. Blood samples were assessed in the NIHR Core Biochemistry Assay Laboratory, Cambridge Biomedical Research Centre for circulating levels of insulin, leptin, glucose, and triglycerides.

To assess circulating insulin, samples were assayed in singleton on a Diasorin Liaison XL automated immunoassay analyzer using a one-step chemiluminescence immunoassay. To assess circulating leptin, an in-house two-site microtitre plate-based DELFIA® assay was used. Triglycerides were measured using an automated colorimetric assay using a Siemens Dimension RxL analyzer. Finally, glucose was assessed using an automatic adaption of the hexokinase-glucose-6-phosphate dehydrogenase method (Kunst et al., 1983) on a Siemens Dimension RxL analyzer.

The Homeostatic model assessment Insulin Resistance (HOMA-IR) model was used to yield an estimate of insulin sensitivity from fasting plasma insulin and glucose concentrations (Matthews et al., 1985). HOMA-IR score was calculated using the following equation: *fasting plasma glucose (mmol/l) X fasting serum insulin (mU/l)*/22.5. There are no established clinical cut-offs for HOMA-IR score (see Gayoso-Diz et al., 2013). As such, to assess the contribution of insulin resistance to memory and brain function, all participants were split by median HOMA-IR score into a high or low insulin resistance groups (see Table 1).

### 2.7 Statistical analysis

Group differences in behavioural performance were assessed using independent samples *t*-tests, and univariate analysis of variance. Pearson's correlations were used to assess associations between brain activity or blood components and memory performance, with Bonferroni correction where multiple comparisons were conducted on the same data.

## 3 Results

### 3.1 Blood components

Group differences in blood components are shown in Table 1. The lean and obese groups differed significantly in their fasting levels of circulating insulin, leptin, and triglycerides, but did not differ in glucose levels. The median HOMA-IR score for the whole sample was 1.94. In order to examine the contribution of insulin resistance to memory and brain activity, the sample of 32 participants was split at this median to create “high” and “low” insulin resistance groupings. This regrouping involved 25% of participants changing groups. When grouped by insulin resistance, the groups differed significantly in insulin and leptin, but not glucose or triglycerides (see Table 1).

### 3.2 Behavioural performance

**Fig. 2:**
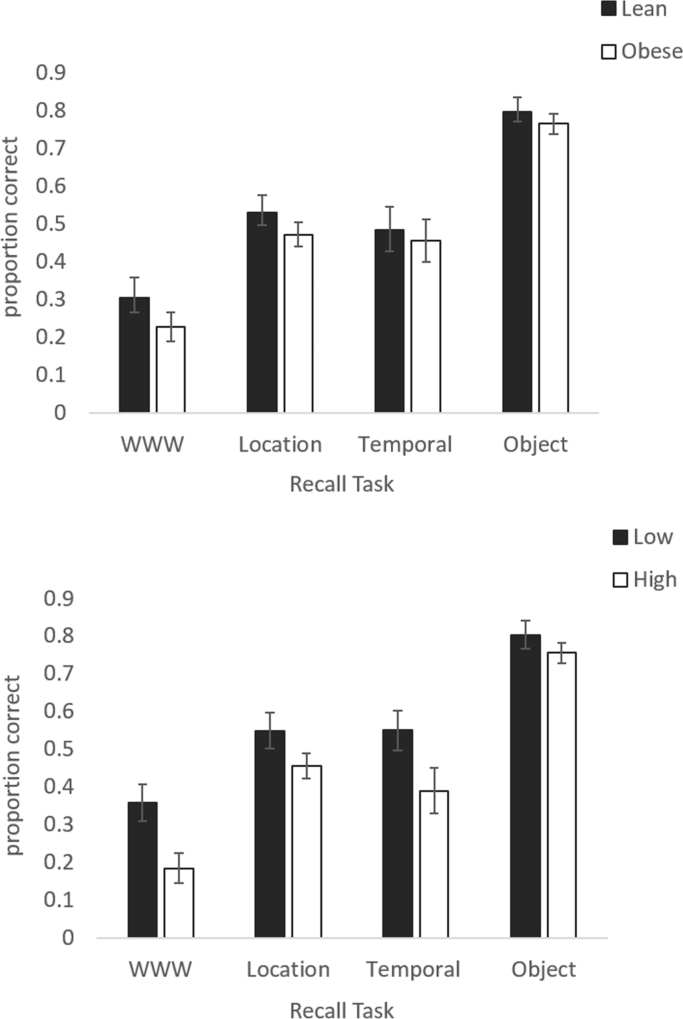
Accuracy in What-Where-When, Location, Temporal and Object retrieval tasks in (top panel) lean and obese participants and (bottom panel) Participants with low and high circulating insulin resistance.

As displayed in Fig. 2, when grouped by BMI, there were no significant differences between lean and obese participants in performance on any of the tasks (WWW t(30)=1.141, p=0.263, d=0.4; Location: t(30)=0.988, p=0.331, d=0.3; Temporal: t(30)=0.330, p=0.744, d=0.1; Object: t(30)=0.472, 343 p=0.640, d=0.2). However, when grouped by IR, participants with higher IR performed significantly more poorly on the WWW task (t(30)=2.632, p=0.013, d=1.0) but not any other task (location t(30)=1.557, p=0.130, d=0.6; temporal: t(30)=2.001, p=0.054,d=0.7; object: t(30)=0.822, p=0.418, d=0.3). The difference between the IR groups in WWW performance appears to be driven by the number of session errors. Individuals with higher IR made significantly more of these errors compared to those with low IR (t(30)=−3.135, p=0.004) but similar numbers of item and spatial errors (item: t(30)=−1.467, p=0.153; spatial: t(30)=−1.458, 350 p=0.155).

## 4 Functional neuroimaging

### 4.1 Neural activity during the Treasure-Hunt task in lean adults

To assess our first hypothesis that the Treasure-Hunt task would be associated with increased activity in regions of interest associated with episodic memory, we first conducted an analysis using only the control (lean) group.

Exploratory whole brain analysis of task-related activity revealed only a single significant activation. An area in the right precuneus (6, −52, 16) was significantly more active during the WWW task than during the control condition (z=4.01; family wise error corrected, p=0.014).

**Fig. 3:**
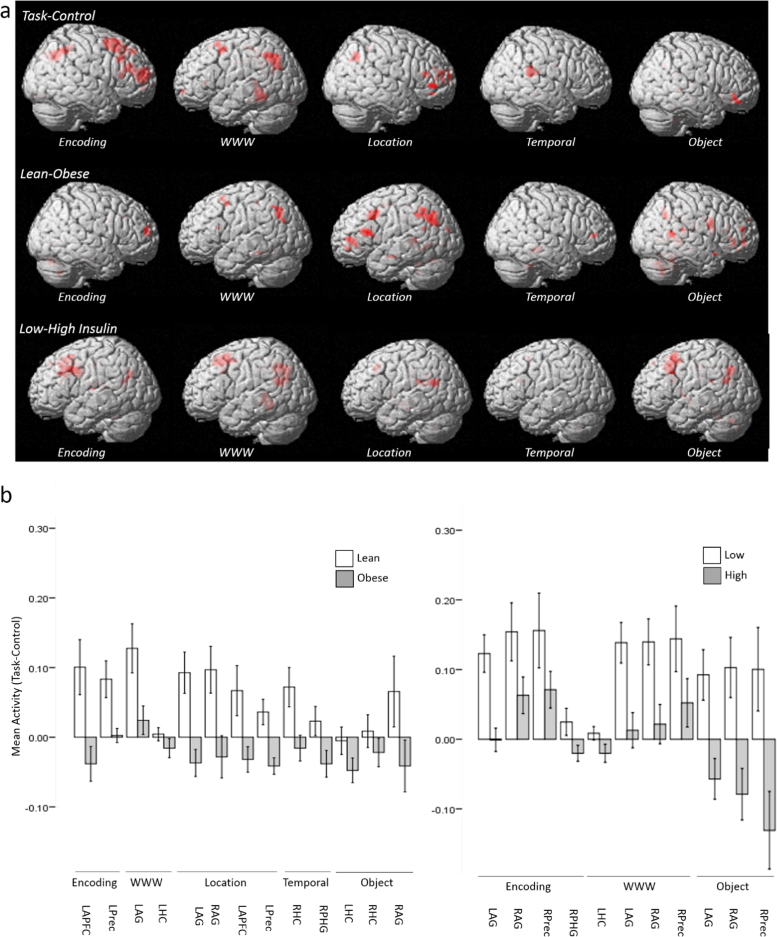
a) (Top Panel): Regions of interest exhibiting greater task-related activity than control during (from left to right) Encoding, WWW, Location, Temporal, Object tasks. (Centre Panel): Regions of interest exhibiting greater task-related activity in lean relative to obese individuals during (from left to right) Encoding, WWW, Location, Temporal, Object tasks. (Bottom Panel): Regions of interest exhibiting greater task-related activity in individuals with low IR relative to those with high IR during (from left to right) Encoding, WWW, Location, Temporal, Object. b) Mean activity levels (Task-Control) across regions of interest in lean and obese participants (left) and high and low IR groups (right).

A priori determined brain regions of interest that exhibited significantly greater activation during the memory tasks than during the control task are shown in Table 3. During both encoding and WWW retrieval, significant activity was observed in the left hippocampus and bilaterally in the angular gyrus (see Fig. 3). Activity was also significantly greater during encoding than the control task in the right dorsolateral PFC and bilaterally in the precuneus. The right angular gyrus and right hippocampus exhibited significant activity during the location and object tasks respectively, compared to the control condition.

### 4.2 Association between functional activity and BMI

To assess our third hypothesis, that obesity would be associated with reduced task-related activity within regions of interest associated with episodic memory, we performed a group level analysis splitting the sample by BMI. Regions of interest that exhibited significantly greater activity in lean compared to obese participants are shown in Table 4. During encoding, group differences were seen in the anterior PFC and left precuneus. During the WWW task, there was a significant group difference in activity in the left angular gyrus. The majority of the group differences were observed during location task performance, in which significantly greater activity was found in the left hippocampus, bilateral angular gyrus, left anterior PFC and left precuneus (Fig. 3). Greater activation was also seen in the right hippocampus and parahippocampal gyrus during the temporal task and bilaterally in the hippocampus and in the right angular gyrus during the object task.

No region of interest exhibited significantly greater activity in obese than lean participants during any task. Addressing whether the observed task-induced activations may have been specific to the regions identified, there were no significant group differences in activity during the control task in any region of interest and no significant group differences in activity in the control area (vertex) during performance of any task.

### 4.3 Association between functional activity and insulin resistance

Finally, to assess the hypothesis that insulin resistance may be related to functional differences as large or larger than those related to BMI, we performed a group level analysis splitting the sample by median insulin HOMA-IR score. Regions of interest that exhibited significantly greater activity in participants with low compared to high IR are shown in Table 5. A rather different pattern of activation was observed compared to when participants were categorized by BMI. During encoding, the low IR group exhibited significantly greater activity bilaterally in the angular gyrus, and in the right parahippocampal gyrus and precuneus. During WWW performance, significantly greater activity was elicited in the low IR group in the right hippocampus, bilaterally in the angular gyrus and in the right precuneus. There were no group differences during the location or temporal task. In the object task the low IR group exhibited greater activation bilaterally in the angular gyrus and in the right precuneus. No region of interest exhibited significantly greater activity in the high IR than in the low IR group during any task. Finally, there were again no significant group differences in activity during the control task in any region of interest and no significant group differences in activity in the control area (vertex) on any task.

There were notably few overlaps between the region/task combinations that differentiated the lean and obese groups and those that differed significantly between the high and low IR groups. This observation suggests that it is doubtful that the relationship between BMI and brain activity is solely driven by group differences in insulin resistance, but that both variables likely play a role in obesity-related memory impairment. Given that group differences in behavioural performance emerged when the sample was split by IR, but not BMI, it is possible that the influence of insulin resistance on brain activity translated more directly to memory performance.

### 4.4 Association between functional activity and task performance

Exploring relationships between brain activity and behavioural accuracy across all participants after Bonferroni correction, activity in the right DLPFC during WWW retrieval correlated significantly with performance on the WWW task (r(32)=0.533, p=0.002, alpha=0.003). This effect seemed to be mediated by integration performance since activity in this area demonstrated a significant negative relationship with session- and item- errors (session: r(32)=−0.635, p<0.001; item: r(32)=−0.616, p<0.001) but no relationship with spatial errors (r(32)=−0.249, p=0.169). Activity in this area during encoding also predicted later item error rate (r(32)=−0.520, p=0.002, alpha=0.006) but not session or spatial errors (session: r(32)=−0.458, p=0.008; spatial: r(32)=0.009, p=0.961). No other correlations between activity and performance were observed in other regions of interest or task.,

### 4.5 Leptin and triglycerides

Leptin correlated with right parahippocampal gyrus activity during temporal memory retrieval (r(32)=−0.457, p=0.008) and with activity in the right angular gyrus (r(32)=−0.419, p=0.018) and left precuneus (r(32)=−0.411, p=0.019) during the WWW task. There were no associations between levels of triglycerides on brain activity during any task. There were no associations between leptin or triglycerides and performance on any task.

## 5 Discussion

To address unresolved questions concerning memory impairment and brain changes in obesity, lean and obese participants matched for age, sex and IQ were scanned using fMRI while performing the Treasure-Hunt episodic memory task. Memory performance was significantly affected by group differences in insulin resistance, but not BMI. Significant activity differences were seen throughout the core recollection network, including the hippocampus, prefrontal cortex, and angular gyrus, with a distinct distribution of activity across these regions when participants were grouped by BMI or insulin resistance. Together, these results add to our understanding of the neural basis of obesity by revealing functional differences in specific areas of the brain associated with recollection.

### 5.1 The neural basis of what-where-when memory

While previous studies have assessed item, spatial and temporal memory separately (e.g. Kwok and Macaluso, 2015) or have assessed object-context integration (e.g. Burgess et al., 2001), the present study represents the first time that an integrated what-where-when task has been characterized using fMRI. This study therefore provides key insights into the neural correlates of these cardinal elements of episodic memory, and how they are integrated (Tulving, 1983). Originating in animal studies of cognition (Clayton and Dickinson, 1998), What-Where-When tests are increasingly used to investigate episodic memory in humans (Cheke, 2016, Cheke and Clayton, 2013, Cheke and Clayton, 2015, Cheke et al., 2016, Easton et al., 2012, Holland and Smulders, 2011). Such tasks, exemplified by the Treasure-Hunt task, are useful because they assess spatial, temporal and item memory within the same paradigm, as well as the ability to integrate these features into a single event recollection. The distinct components of the Treasure-Hunt task were associated with activity in a variety of areas within the core recollection network, and broadly corresponded to areas activated in previous studies of individual elements (Burgess et al., 2001; Kwok and Macaluso, 2015). In particular, elements of the task that involved integration of location, object, and temporal information (i.e. encoding and WWW retrieval), rather than the components that did not require integration (location, temporal, and object retrieval), elicited activity within the left hippocampus and left angular gyrus. Both regions have been previously implicated in the multimodal integration of distributed episodic features into a coherent memory representation (Burgess et al., 2002; Shimamura, 2011; Yazar et al., 2012; Yazar et al., 2014). These findings suggest that the Treasure-Hunt task successfully differentiates memory processes that do and do not require integration, while holding the content of that memory constant. It is possible that some differences in activity across tasks could have resulted from differences in arousal produced by the use of a block presentation. However, given the complexity of the different tasks, a block design was necessary to maintain performance, and the order of the tasks were strictly counterbalanced to account for possible time- related changes in arousal.

The right dorsolateral prefrontal cortex (RDLPFC) and the precuneus (bilaterally) were significantly more active than in the control condition during encoding but not during any of the retrieval tasks. Both areas have previously been associated with the encoding of visuospatial information and of relationships between items (Frings et al., 2006, Murray and Ranganath, 2007). Given the established role of RDLPFC in retrieval monitoring and the self-organization of responses (D'Esposito et al., 1999, McDonough et al., 2013, Petrides et al., 1993a, Petrides et al., 1993b), activity in this region may reflect the maintenance of what-where-when associations online to facilitate encoding strategies. This interpretation is supported by the finding that activity in the RDLPFC in both encoding and retrieval correlated significantly with accuracy on the WWW task.

### 5.2 BMI, memory, and brain activity

The main aim of this study was to assess the association between obesity and memory ability, both behaviourally and in terms of neural activity. In general, the term “obesity” is defined using body mass criteria. When functional activity was contrasted between lean and obese individuals classified by BMI, group differences were found throughout the core recollection network, and across most tasks. This was in contrast to a control area (vertex) and a control task, in which there were no significant group differences, perhaps suggesting that the observed task-induced activity may have been specific to core recollection regions. The hippocampus exhibited significantly less activity in obese individuals during the location, temporal and object control tasks, but not during encoding or the WWW integration task. The left angular gyrus was significantly less active in obese individuals during the WWW task but not during any of the other tasks. Both the hippocampus and angular gyrus are implicated in the integrating episodic features into a coherent representation and the angular gyrus in particular has been commonly associated with memory vividness (Shimamura, 2011, Yazar et al., 2012, Yazar et al., 2014). Patients with lesions to the angular gyrus (or individuals with temporary lesions induced by transcranial magnetic stimulation) tend not to have deficits in memory accuracy, but report reduced memory vividness and confidence (Davidson et al., 2008, Yazar et al., 2014). This raises the question of whether obese individuals may experience memory less vividly than their lean counterparts. This cannot be assessed with the current data and therefore requires further investigation, but if true would be of particular significance given evidence that the vividness of meal-memories may play a key role in regulating consumption (Higgs, 2008).

Both the encoding and location tasks elicited between-group differences in left anterior PFC activity. Activity in this region has been previously related to monitoring and evaluation of specific memory characteristics, and particularly object-location associations, at retrieval (Burgess et al., 2001; Ranganath et al., 2000). This region is also associated with process and sub-goal selection, and with prospective memory (Burgess et al., 2000; Fletcher and Henson, 2001) and may therefore be related to the maintenance and execution of strategies during encoding. These findings are in line with the considerable literature on executive function deficits associated with obesity (Barkin, 2013, Elias et al., 2003) and may suggest that obese individuals may be less able to monitor specific characteristics to ensure accurate memory retrieval. Targeted future research would be required to investigate this idea.

One possible mechanism by which obesity may lead to cognitive deficits is via alterations in circulating leptin. Released by adipose tissue, leptin is present in much higher concentrations in obese individuals, often leading to leptin resistance (Enriori et al., 2006) which has been associated with cognitive deficits (e.g. Harvey, 2007). Here, levels of circulating leptin were most strongly associated with reduced activity in the right parahippocampal gyrus during temporal memory retrieval. This was also an area that was significantly less activated in obese compared to lean participants. However, given that the other area/task combinations that differed between lean and obese individuals did not show a relationship with leptin, and that those task/area combinations that did show an association with leptin did not differ between the lean and obese groups, it would seem unlikely that leptin is a key mediator of the reduced activity seen in obese individuals.

Given group differences in neural activity, alongside previous findings of a negative association between BMI and Treasure-Hunt task performance (Cheke et al., 2016), it was surprising that the BMI groups did not show significantly different memory accuracy. Activity effects in the absence of behavioural differences may reflect sub-threshold impairments that alter neural processing without yet impacting on performance, or may indicate reductions in factors that do not themselves directly impact behavioural accuracy. For example, impairments in the parietal lobe (and in the angular gyrus in particular) are rarely associated with reductions in memory accuracy, but rather with memory vividness and confidence (Yazar et al., 2014).

There are a number of possible reasons for the discrepancy in behavioural findings between the present study and Cheke and colleagues’ study. Performance was in general considerably lower in this study compared to previous studies using the Treasure-Hunt task. This is a common finding when conducting cognitive tasks within an MRI scanner and may have reduced variability in the sample, especially given that the scan was conducted early in the morning in participants who had gone without breakfast. Considerable evidence suggests that obesity may be associated with reduced IQ (Goldberg et al., 2014, Olsson and Hulting, 2010, Volkow et al., 2009). By strictly controlling for group differences in IQ in the present study, we may have minimized IQ-related memory differences that typically distinguish lean and obese populations. Another possibility is that the behavioural effect observed by Cheke and colleagues was in fact driven by variance in insulin sensitivity, which were not assessed in that study.

### 5.3 Insulin resistance, memory, and brain activity

Insulin resistance has been suggested as a key factor driving neural deficits in obesity (e.g. Lamport et al., 2014), and it was therefore predicted that HOMA-IR score, which offers an estimate of insulin sensitivity, would reflect memory impairments and neural activity, potentially to a greater degree than BMI. To investigate this, participants were split into “high” and “low” IR groups regardless of BMI. When grouped this way, significant group differences in memory performance were found in the WWW task. Grouping by insulin resistance produced a pattern of functional activity differences similar but distinct from that observed when comparing BMI groups. Interestingly, differences were seen in the same regions (e.g. hippocampus, angular gyrus, precuneus) but during different tasks. This suggests that BMI and insulin may impact on the same brain regions but affect different processes. Given the behavioural findings, it may be that those processes affected by insulin have a closer association with memory accuracy than those affected by BMI. Unfortunately, because of the high level of association between BMI and insulin (75%) it was not possible to investigate group by region by task interactions directly or to investigate the relative contribution of each variable to memory performance.

This study has a number of limitations. The limited sample and degree of overlap between variance in adiposity and insulin resistance makes it very difficult to assess the relative contribution of these factors to memory performance and memory-related brain activity. Future studies would benefit from a more extensive sample specifically recruited to have better independence between adiposity and insulin sensitivity. It is possible that one issue contributing to lack of clarity in this area is the use of BMI as a measure of adiposity. While it is, by some distance, the most commonly used measure in this field, BMI is vulnerable to confounds from muscle and bone density, and as such future studies would benefit from using more sophisticated measures of adiposity such as percent body-fat or visceral fat. A further possible confound lies in the use of food items as stimuli, for these may have induced differential levels of neural response in lean and obese participants. However, evidence suggests that food images produce, if anything, greater activation in core recollection network areas in obese participants (Martin et al., 2010) and would therefore not predict the pattern of neural activity seen in the present study.

## 6 Conclusions

The results presented here provide the first evidence for regionally specific reduced brain activity in obese individuals during episodic memory task performance. This builds on previous findings that obesity is associated with episodic memory impairments (Cheke et al., 2016), and that overweight individuals show functional and structural reductions within the core recollection network when engaged in non-memory tasks (Willette and Kapogiannis, 2015). The present findings suggest that obesity is related to functional changes in the memory areas of the brain, and that insulin resistance may be a key player in this association.

## Figures and Tables

**Table 1.** **Participant (N=32) demographics when grouped by BMI (left) or insulin resistance (right)**.

	**Mean (SD)**		***Group differences***	**Mean (SD)**		***Group differences***
	**Range**			**Range**		
	**Lean group (BMI<25)**	**Obese group (BMI>30)**		**Low IR group (<1.940)**	**High IR group (>1.940)**	
Sex (M/F)	9/7	9/7	χ^2^=0, p=1	10/6	8/8	χ^2^=0.508, p=0.476
Age (years)	27.3 (5.9)	27.7 (5.7)	t(30)=−0.181, p=0.857	26.7 (5.9)	28.3 (5.6)	t(30)=−0.793, p=0.434
				19–35	18–35	
	18–34	20–35				
BMI (kg/m^2^)	21.29 (2.1)	34.29 (4.0)	t(30)=11.491, p<0.001	23.73 (5.2)	31.85 (7.0)	t(30)=3.3734, p=0.001
				18–32	20–47	
	18–25	29–47				
IQ (Ravens)	0.6024 (0.2)	0.5104 (0.2)	t(30)=1.114, p=0.274	0.6215 (0.2)	0.4913 (0.2)	t(30)= 1.610, p=0.118
				0.22–0.94	0.14–1	
	0.139–0.944	0.194–1.0				
Insulin (pmol/l)	38.23 (19.3)	86.4 (47.8)	t(30)= 3.735, p=0.001	30.55 (9.8)	94.08 (40.6)	t(30)=6.08, p<0.001
	13.9–90.5	25.6–171.5		13.9–46.6	51.2–171.5	
HOMA-IR	1.48 (0.75) 0.55–3.46	3.47 (1.91) 0.96–6.71	t(30)=3.863, p=0.001	1.17 (0.37) 0.55–1.88	3.77 (1.61) 2.0–6.71	t(30)=6.286, p<0.001
Leptin(ng/ml)	9.26 (7.5)	41.19 (28.4)	t(30)=4.341, p<0.001	14.81 (16.6)	35.65 (30.1)	t(30)=−2.429, p=0.021
	0.2–22.9	5.9–88.7		0.2–63.6	2.8–88.7	
Glucose(Mmol/l)	5.24 (0.38)	5.42 (0.31)	t(30)=1.471, p=0.152	5.22 (0.34)	5.44 (0.34)	t(30)=−1.806, p=0.081
	4.6–6	5–6.1		4.6–6	4.9–6.1	
Triglycerides (mmol/l)	0.63 (0.26)	0.86 (0.24)	t(30)=2.649, p=0.013	0.65 (0.25)	0.83 (0.27)	t(30)=−1.988, p=0.056
	0.2–1.1	0.5–1.3		0.2–1.1	0.3–1.3	

**Table 2.** **Regions of interest**.

**Study**	**Task**	**Region**	**MNI Coordinates**
Kuhn and Gallinat (2014)	Overlap between areas sensitive to spatial navigation and episodic memory	Hippocampus (bilateral)	−18, −34, −5
			22, −34, 7
Vilberg and Rugg (2008)	Area in parietal lobe reliably more associated with recollection than familiarity	Angular Gyrus (bilateral)	−43, −66, 38
			43, −66, 38
Burgess et al. (2001)	More active when retrieving object-location associations than when answering perceptual questions	Anterior Prefrontal Cortex (bilateral)	−27, 51, −3
			30, 57, 3
		Ventrolateral Prefrontal Cortex (bilateral)	−27 24 29
			30 24 26
		Parahippocampal Gyrus (right)	24, −33, −18
		Precuneus (right)	3, −69, 24
Frings et al. (2006)	Encoding and recognition of spatial relations	Precuneus (left)	−15, −66, 30
Sommer et al. (2005)	Encoding of object-location associations	dorsolateral PFC (right)	51 36 27
Jung et al., 2016, Okamoto et al., 2004	Control area	Vertex	0 −15 74

**Table 3:** Regions demonstrating significantly greater activation during task than control in lean participants.

**Contrast**	**Brain area**	**Hemisphere**	**Z**	**P (FWE)**	**Peak MNI coordinates (x,y,z)**
ENCODING-CONTROL	Hippocampus	Left	3.00	0.040	−27, −40, 4
	Angular Gyrus	Left	3.13	0.029	−36, −70, 40
		Right	3.48	0.011	42, −61, 40
	Dorsolateral Prefrontal cortex	Right	3.32	0.017	51, 35, 22
	Precuneus	Right	3.24	0.022	0, −70, 38
		Left	3.07	0.034	−12, −67, 30
WWW-CONTROL	Hippocampus	Left	3.36	0.021	−21, −40, −2
	Angular Gyrus	Left	3.65	0.010	−42, −61, 44
		Right	3.50	0.015	42, −61, 44
LOCATION-CONTROL	Angular Gyrus	Right	3.25	0.029	42, −61, 40
TEMPORAL-CONTROL	No Clusters				
OBJECT-CONTROL	Hippocampus	Right	3.61	0.009	21, −37, 2

**Table 4.** Regions that demonstrated significantly greater activity in lean than obese participants.

**Contrast**	**Brain area**	**Hemisphere**	**Z**	**P (FWE)**	**Peak MNI coordinates (x,y,z)**
ENCODING-CONTROL	Anterior Prefrontal cortex	Left	3.10	0.034	−27, 53, −4
	Precuneus	Left	3.04	0.039	−18, −67, 28
WWW-CONTROL	Angular Gyrus	Left	3.19	0.033	−39, −64, 36
LOCATION-CONTROL	Hippocampus	Left	3.51	0.012	−24, −40, 0
	Angular Gyrus	Left	3.65	0.008	−42, −67, 34
		Right	3.37	0.019	42, −58, 40
	Anterior Prefrontal cortex	Left	3.08	0.042	−33, 50, −6
	Precuneus	Left	3.51	0.012	−18, −64, 30
TEMPORAL-CONTROL	Hippocampus	Right	3.20	0.027	18, −37, −10
	Parahippocampal gyrus	Right	3.10	0.036	−21, −37, −10
OBJECT-CONTROL	Hippocampus	Left	3.04	0.041	−18, −34, 6
		Right	3.78	0.005	21, −40, 2
	Angular Gyrus	Right	3.23	0.024	39, −58, 38

**Table 5.** Regions that demonstrated significantly greater activity in participants with low than high fasting insulin levels.

**Contrast**	**Brain area**	**Hemisphere**	**Z**	**P (FWE)**	**Peak MNI coordinates (x,y,z)**
ENCODING-CONTROL	Angular Gyrus	Left	3.07	P=0.038	−42, −67, 34
		Right	3.20	P=0.026	48, −64, 36
	Precuneus	Right	2.96	P=0.05	9, −61, 20
	Parahippocampal Gyrus	Right	2.96	P=0.05	9, −61, 30
WWW-CONTROL	Hippocampus	Right	3.77	P=0.019	15, −43, −6
	Angular Gyrus	Left	3.11	P=0.042	−39, −64, 40
		Right	3.72	P=0.007	45, −64, 36
	Precuneus	Right	3.41	P=0.018	9, −61, 30
LOCATION-CONTROL	No Clusters				
TEMPORAL-CONTROL	No Clusters				
OBJECT-CONTROL	Angular Gyrus	Left	3.40	P=0.015	−39, −67, 38
		Right	3.29	P=0.021	45, −61, 38
	Precuneus	Right	3.54	P=0.01	6, −61, 30
